# R-loops: a key driver of inflammatory responses in cancer

**DOI:** 10.1038/s12276-025-01495-0

**Published:** 2025-07-08

**Authors:** Seo Yun Lee, Min Jae Kwak, Jae Jin Kim

**Affiliations:** Department of Life Science and Multidisciplinary Genome Institute, Hallym University, Chuncheon, Republic of Korea

**Keywords:** Biological sciences, Genetics

## Abstract

R-loops, which are noncanonical three-stranded nucleic acid structures formed when RNA hybridizes with complementary DNA strand while displacing the other DNA strand, have emerged as crucial players in cellular homeostasis and cancer pathogenesis. Here we explore the intricate relationship between R-loops and inflammation in the context of cancer development and progression. R-loops can trigger inflammatory responses through various mechanisms, including DNA damage induction, genome instability and activation of innate immune pathways, particularly in cancer cells, where R-loop regulation is frequently dysregulated. In the tumor microenvironment, R-loop-mediated genomic instability contributes to inflammatory signaling cascades, affecting both cancer cells and the surrounding tumor microenvironment. We discuss how aberrant R-loop formation influences key inflammatory pathways, including the cGAS–STING axis and NF-κB signaling, and their subsequent effects on tumor progression. Furthermore, we explored how cancer cells manipulate R-loops to modify their inflammatory microenvironment, potentially affecting their therapeutic responses. Understanding the complex interplay between R-loops and cancer-associated inflammation provides novel insights into tumor biology and opens new avenues for therapeutic intervention. This Review summarizes the current knowledge on R-loop biology in cancer, its inflammatory consequences and potential strategies for targeting R-loop-mediated inflammation in cancer treatment, underscoring the importance of this emerging field in cancer medicine.

## R-loops drive inflammation and cancer progression insights

Cells constantly undergo changes, and one such change involves R-loops. R-loops are three-stranded structures formed when RNA binds to DNA, leaving one DNA strand single. While R-loops are normal and help in various cell functions, their imbalance can cause problems such as cancer. This Review explores how R-loops can both promote and fight cancer. Researchers explored how R-loops affect cancer by either causing instability in the genome or triggering immune responses. They used various methods to study these effects, focusing on how R-loops interact with immune pathways such as cGAS–STING, which helps the body in fighting tumors. The study found that, while excessive R-loops can lead to cancer by causing DNA damage, they can also activate immune responses that help to fight cancer. The researchers suggest that targeting R-loop pathways could be a new way to treat cancer.

This summary was initially drafted using artificial intelligence, then revised and fact-checked by the author.

## Introduction

Cellular genomes are constantly subjected to various structural alterations, among which R-loops have emerged as important regulators of both physiological and pathological processes^1,2^. R-loops are three-stranded nucleic acid structures that are formed when RNA hybridizes with a complementary DNA strand, displacing the other DNA strand into a single-stranded conformation^3^. While these structures naturally occur during transcription and play essential roles in various cellular processes, their dysregulation can lead to genomic instability and cellular stress^4–7^.

The role of R-loops in cancer presents an intriguing paradox. On the one hand, excessive R-loop formation can lead to genomic instability, potentially promoting tumor initiation and progression^8–11^. On the other hand, R-loop-mediated activation of innate immune responses, particularly through the cGAS–STING pathway, can trigger antitumor immunity^12–14^. This dual nature of R-loops in cancer reflects the complex relationship between genomic stress responses and immune surveillance in tumor biology.

The immune response activated by R-loops primarily involves the production of type I interferons and pro-inflammatory cytokines, which can enhance immune cell recruitment and activation. This Review aims to provide a comprehensive overview of the dual role of R-loops in cancer, with particular emphasis on their immunostimulatory effects and the mechanisms by which cancer cells respond to and modulate these responses. We will examine the molecular pathways linking R-loop formation to immune activation, explore how different cancer types handle R-loop-induced immune responses and discuss the therapeutic implications of targeting R-loop biology for cancer treatment.

## R-loop formation and resolution

The proper formation and resolution of R-loops are crucial for maintaining multiple cellular processes. R-loops serve numerous essential physiological functions in transcription regulation, acting far beyond mere byproducts of transcription. At CpG island promoters, they establish and maintain open chromatin states conducive to transcription initiation while preventing DNA methylation, particularly at housekeeping genes^15^. R-loops modulate gene expression by influencing RNA polymerase II elongation rates, which affects alternative splicing and non-coding RNA expression^16^. R-loops are integral to immunoglobulin class switch recombination, enabling antibody diversification, and contribute to DNA replication by serving as primers at certain origins^17,18^. They participate in DNA repair pathways and help to maintain genome stability at telomeres and centromeres^9,19,20^. In addition, R-loops influence chromatin modification patterns and epigenetic regulation by recruiting specific protein complexes^21^. While these functions demonstrate the beneficial aspects of R-loops, their precise regulation is critical, as dysregulation can lead to genomic instability and various pathological conditions^5^.

**Fig. 1:**
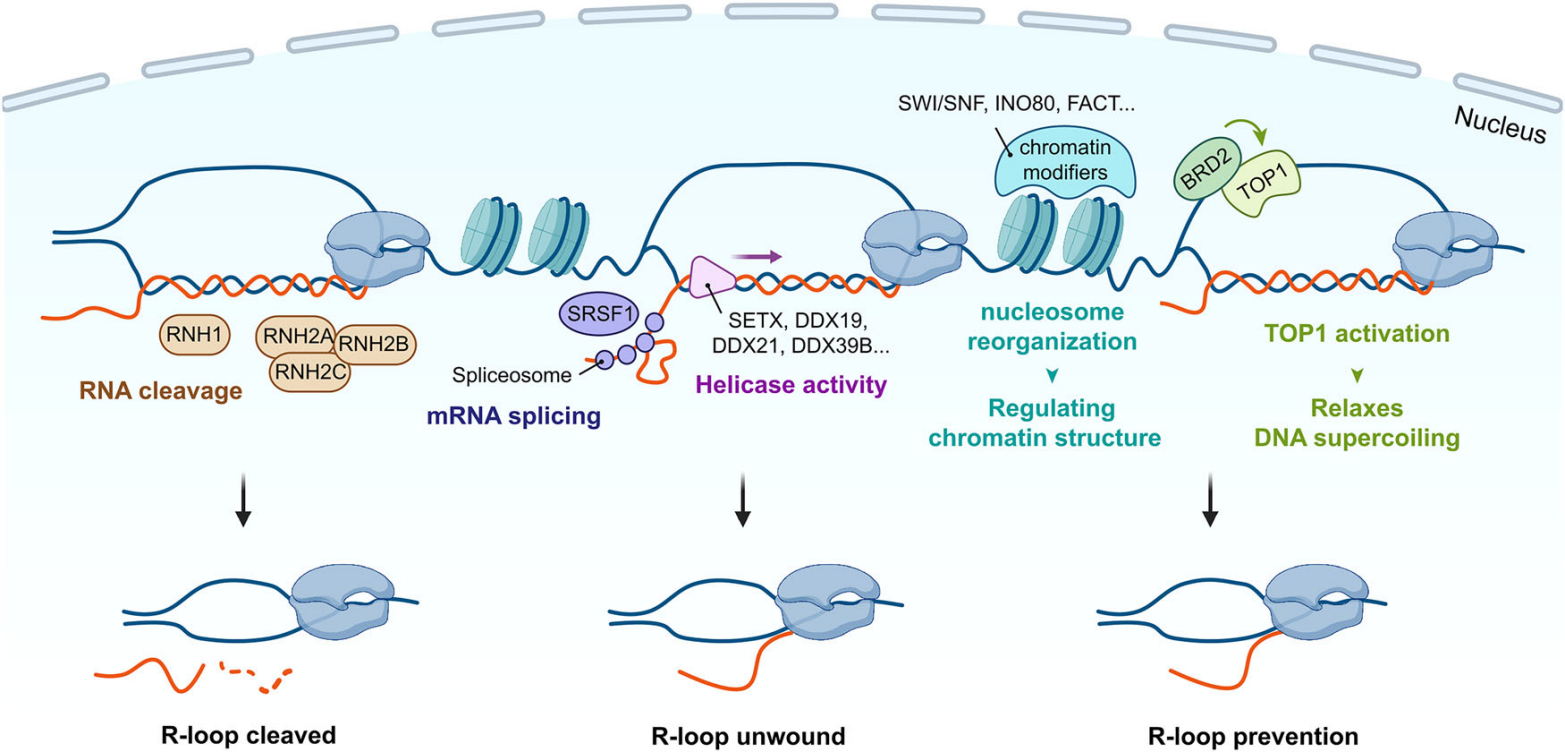
Molecular mechanisms regulating R-loop homeostasis. Various cellular factors coordinate to prevent and resolve R-loop formation during transcription. RNase H enzymes (RNaseH1 and RNaseH2A/B/C) directly cleave the RNA strand of RNA–DNA hybrids, thereby resolving R-loop structures; Splicing factors such as SRSF1 and THO complexes regulate co-transcriptional mRNA processing, while helicases including SETX, DDX19, DDX21 and DDX39B unwind RNA–DNA hybrids to prevent aberrant R-loop persistence; Chromatin remodelers (for example, SWI/SNF, INO80 and FACT) and histone modifiers dynamically reorganize nucleosome and modulate chromatin accessibility, thereby mitigating transcription-induced DNA supercoiling. The PRC1 complex and SIN3A suppress R-loop accumulation through histone modifications, whereas KAT8-mediated histone H4 acetylation facilitates the recruitment of BRD2 and BRD4. BRD2, in turn, promotes TOP1 activity to resolve DNA supercoiling, preventing R-loop formation during active transcription.

Aberrant R-loop accumulation is tightly regulated in cells. Various factors are involved in this regulation, including RNase H enzymes, RNA processing factors (such as splicing proteins and export factors), helicases (for example, SETX, DDX19, DDX21 and DDX39B), chromatin modifiers and topoisomerase I (TOP1) (Fig. 1). Dysregulation of these factors can lead to excessive R-loop accumulation, which can result in genomic instability. A major source of this genomic instability arises from transcription–replication conflicts (TRCs), where the transcription and replication machineries collide^22,23^. R-loops formed during transcription can impede the progression of replication forks, leading to fork stalling or collapse. These conflicts are particularly problematic in highly transcribed regions or in cells with defects in R-loop resolution mechanisms. When persistent, TRC-associated R-loops can trigger DNA double-strand breaks, chromosomal rearrangements and other forms of genomic instability that contribute to cancer initiation and progression.

At the forefront of R-loop resolution are the RNase H enzymes, RNaseH1 and RNaseH2^24,25^. These specialized enzymes specifically cleave the RNA strand in RNA–DNA hybrids, effectively dismantling the R-loop structure. RNaseH1 operates in both the nucleus and mitochondria^26^, while RNaseH2 is confined to the nucleus and interacts with PCNA, potentially aiding its recruitment to sites of DNA replication and repair^27–29^. In addition to RNase H enzymes, several RNA–DNA helicases play crucial roles in unwinding R-loops. Senataxin (SETX) is particularly important for resolving R-loops at transcription termination sites and double-strand breaks^30–32^. Other helicases, including DHX9^33–35^, DDX19^36^, DDX21^37^ and DDX39B^38^, also contribute to R-loop resolution in various cellular contexts. At telomeres, helicases such as BLM^39^ and FANCM^40,41^ are specifically involved in unwinding telomeric R-loops. The process of transcription itself incorporates mechanisms to prevent and resolve R-loops. RNA processing factors, such as splicing proteins (SRSF1, SFPQ and SF3B1) and the TREX complex^42–46^, help to prevent nascent RNA from reannealing to the DNA template and promote mRNA export, respectively. Topoisomerases, particularly TOP1, resolve DNA supercoiling during transcription and replication, which can prevent R-loop formation^47,48^. Chromatin structure and modification also play roles in R-loop resolution. Chromatin remodeling complexes such as SWI/SNF, INO80 and FACT aid in resolving R-loops by reorganizing chromatin structure^49–51^. Histone modifiers, including histone deacetylases such as the SIN3A complex and acetylation readers such as BRD2 and BRD4, can suppress R-loop formation through their effects on Pol II and TOP1^52–55^. A recent study identified that PCAF, a histone acetyltransferase, suppresses R-loop formation through H4K8ac, which recruits MRE11 and EXO1 to R-loop sites^56^.

DNA repair pathways are intricately linked with R-loop resolution. Homologous recombination factors, notably BRCA1 and BRCA2, are involved in both suppressing and resolving R-loops, particularly at sites of DNA damage^32,57–59^. The Fanconi anemia pathway proteins (FANCD2, FANCI and FANCA) also play crucial roles in R-loop suppression and resolution, coordinating these processes with replication and transcription^60–62^.

## R-loop-mediated inflammation

**Fig. 2:**
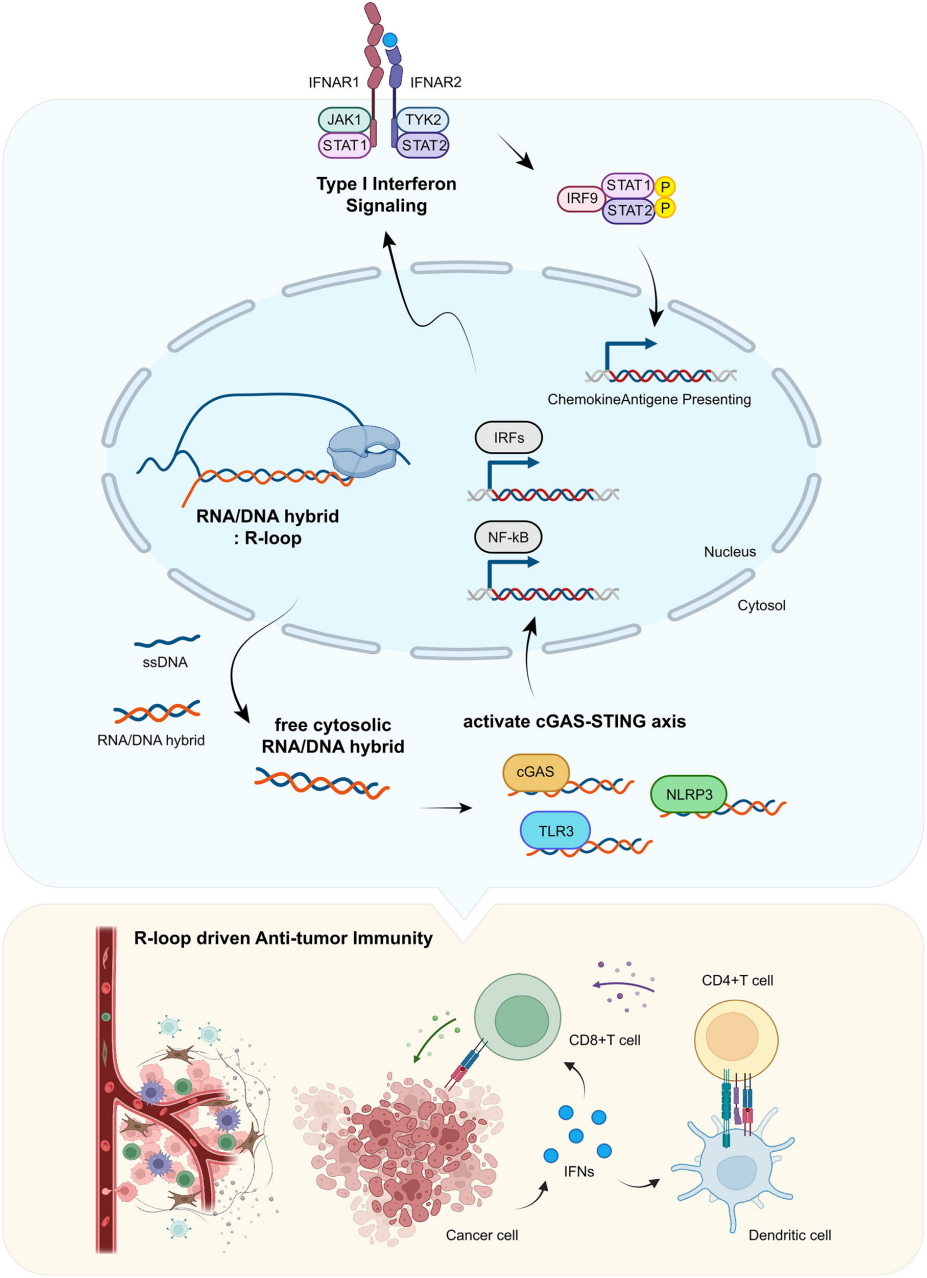
R-loop-mediated antitumor immunity. R-loops trigger antitumor innate immune responses through type I IFN signaling pathways. Top: activation of type I IFN signaling by R-loops. In the nucleus, accumulated R-loops lead to the release of RNA–DNA hybrids into the cytosol, which are recognized by various immune sensors including cGAS, TLR3 and NLRP3. These signals activate the cGAS–STING pathway and cascade downstream signaling through IRFs and NF-kB transcription factors. Bottom: the resulting IFN production promotes antitumor immunity by activating CD8^+^ and CD4^+^ T cells, and dendritic cells, ultimately enhancing the R-loop-driven immune responses.

These resolution factors play crucial regulatory roles in maintaining R-loop homeostasis, and mutations in these factors can have severe consequences^14^. When these factors are mutated or dysfunctional, cells experience abnormal accumulation of R-loops, leading to increased genomic instability and DNA damage. This DNA damage process generates R-loop-derived nucleic acids, which can include DNA fragments, RNA–DNA hybrids and other nucleic acid species. These nucleic acid fragments serve as molecular signals that are recognized by various cellular sensors. The cellular response to R-loops involves sophisticated mechanisms for detecting and responding to these unique nucleic acid structures. Various pattern recognition receptors (PRRs) have evolved to recognize different aspects of R-loop-associated nucleic acids, leading to the activation of inflammatory signaling pathways that can impact cancer development and progression^13^ (Fig. 2).

Among the PRRs, Toll-like receptors play a fundamental role in detecting R-loop-derived nucleic acids. TLR3 recognizes double-stranded RNA and RNA–DNA hybrids^14,63^, TLR7/8 detects single-stranded RNA, and TLR9 recognizes DNA in endosomal compartments^64,65^. Among these nucleic-acid-sensing TLRs, TLR3 is particularly important as it has been specifically identified to bind RNA–DNA hybrids and R-loops^14^. TLR3, primarily expressed in dendritic cells and various tissue cells, plays a fundamental role in nucleic acid sensing. While traditionally known for recognizing viral double-stranded RNA in endosomal compartments, TLR3 has also been shown to detect RNA–DNA hybrids.

The cGAS–STING pathway represents another critical mechanism for detecting cytoplasmic RNA–DNA hybrids resulting from R-loop-induced genomic instability^14^. When cytoplasmic RNA-DNA hybrid is detected by cGAS, it triggers the production of cGAMP, which serves as a second messenger molecule. cGAMP then binds to and activates STING at the endoplasmic reticulum membrane^66^. Upon activation, STING undergoes trafficking to the Golgi apparatus, where it recruits and activates TBK1. This process ultimately leads to the activation of IRF3, which translocates to the nucleus and induces the expression of interferon (IFN)-stimulated genes and various chemokines. In addition, activated STING can trigger NF-κB signaling, further amplifying the inflammatory response^67^. Inflammasomes represent another layer of nucleic-acid-sensing machinery that can respond to R-loop-derived structures. Several inflammasome complexes, including NLRP3, AIM2 and IFI16, can assemble in response to nucleic acids in the cytoplasm^68,69^. These macromolecular signaling complexes typically contain a sensor, an adapter and an effector component. When activated, inflammasomes trigger the production of pro-inflammatory cytokines, particularly IL-1β and IL-18, and can induce inflammatory cell death^67^. The presence of multiple, sophisticated mechanisms for detecting and responding to R-loop-derived nucleic acids underscores the biological importance of these structures in cellular homeostasis and disease. In the context of cancer, the integration of signals from these various pathways can create complex feedback loops that influence the tumor microenvironment. Understanding how these pathways interact and regulate each other remains crucial for developing effective therapeutic strategies targeting R-loop-mediated inflammation in cancer.

## Cellular mechanisms of R-loop-mediated inflammation in the tumor microenvironment

The production of type I IFNs through R-loop-mediated signaling pathways profoundly reshapes the tumor microenvironment^70,71^. When R-loop-derived nucleic acids activate the cGAS–STING axis or TLR3 signaling, the resulting type I IFN production initiates a cascade of events that remodels the immune landscape within and surrounding the tumor^72–74^ (Fig. 2). Type I IFNs (primarily IFN-α and IFN-β) bind to the heterodimeric receptor complex composed of IFNAR1 and IFNAR2 on target cells, activating the JAK–STAT signaling pathway^75,76^. This leads to the phosphorylation of STAT1 and STAT2, which form complexes with IRF9 (known as ISGF3) and translocate to the nucleus. There, they induce the expression of hundreds of IFN-stimulated genes (ISGs), including those encoding chemokines and antigen-presenting machinery, thus amplifying the immune response.

In the context of antitumor immunity, these type I IFNs serve as critical bridges between innate and adaptive immune responses. They enhance the cytotoxic activity of CD8^+^ T cells^77,78^ and natural killer (NK) cells^79,80^, improving their capacity to recognize and eliminate cancer cells. Concurrently, dendritic cells exposed to type I IFNs undergo maturation, increasing their antigen presentation capabilities and promoting the cross-presentation of tumor-associated antigens to CD8^+^ T cells^81–84^. This process is essential for priming effective antitumor T cell responses. In addition, type I IFNs induce the expression of MHC class I molecules on cancer cells, making them more visible to the immune system^85^.

The chemokine profile induced by type I IFNs also plays a crucial role in recruiting immune cells to the tumor site^86,87^. CXCL9, CXCL10 and CXCL11, chemokines induced by IFN signaling, attract CXCR3-expressing T cells and NK cells to the tumor microenvironment. This infiltration of cytotoxic lymphocytes is strongly associated with favorable outcomes in multiple cancer types. However, the relationship between R-loop-induced type I IFNs and cancer progression is complex^88^. Chronic IFN signaling can lead to immunosuppression through various mechanisms, including the upregulation of immune checkpoint molecules such as PD-L1 on tumor cells and the recruitment of immunosuppressive cell populations. Moreover, cancer cells can adapt to persistent type I IFN signaling by developing resistance mechanisms that allow them to evade immune surveillance while benefiting from certain pro-survival effects of IFN signaling.

The temporal dynamics of type I IFN signaling are also critical in determining outcomes. Acute, robust IFN responses typically promote antitumor immunity, while chronic, low-level signaling may support tumor growth and metastasis^89^. This dichotomy explains why IFN-related gene signatures have been associated with both favorable and unfavorable prognoses in different cancer contexts.

## R-loop-mediated inflammation in hematological malignancies

**Fig. 3:**
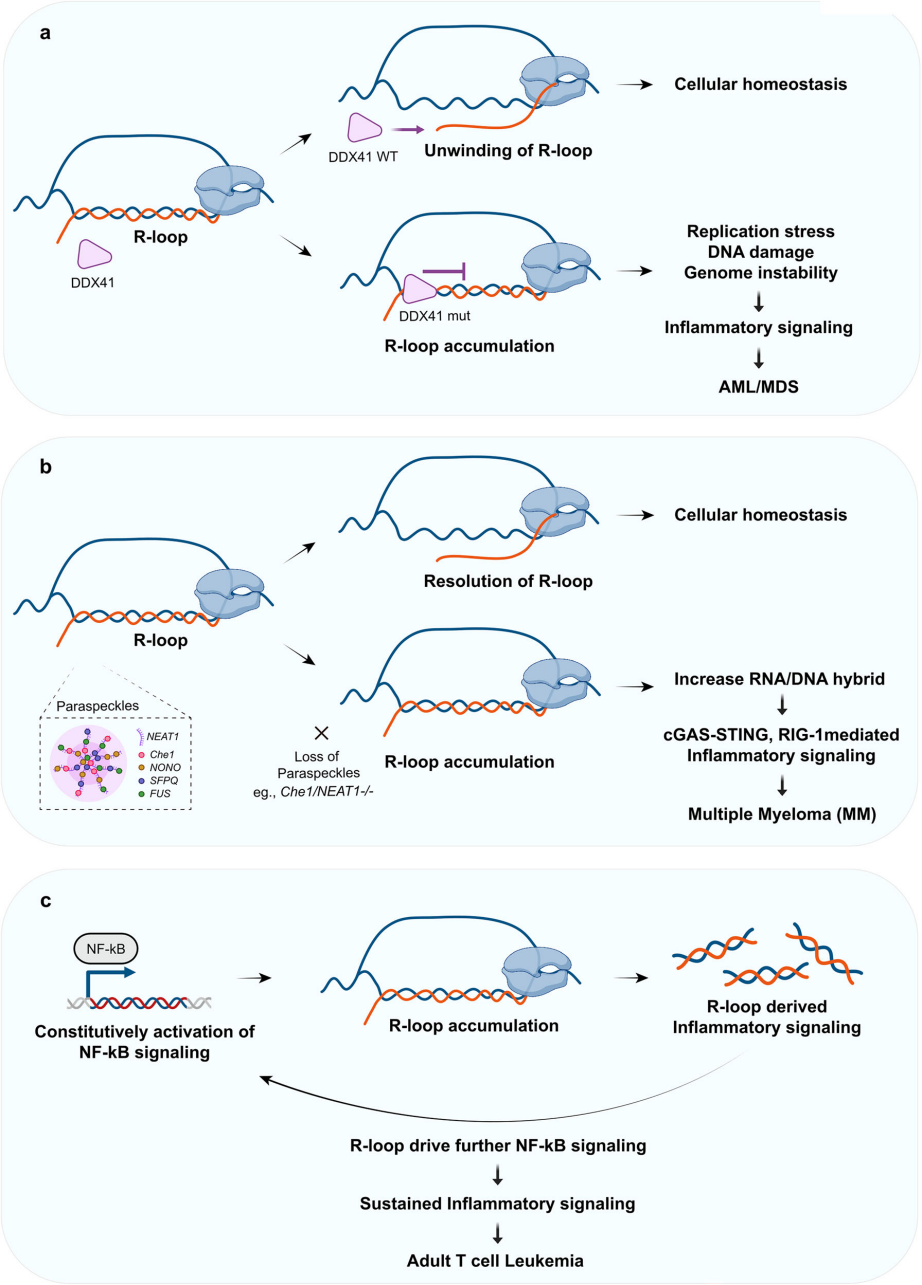
R-loop-mediated inflammatory signaling in hematological malignancies. **a** DDX41-dependent regulation of R-loops. Wild-type DDX41 properly unwinds R-loops, maintaining cellular homeostasis. However, mutant DDX41 leads to R-loop accumulation, triggering replication stress, DNA damage and genome instability, ultimately contributing to AML or MDS development through inflammatory signaling. **b** Paraspeckle-dependent R-loop regulation in multiple myeloma. Paraspeckles, nuclear bodies containing various factors including NEAT1, Che-1, NONO, SFPQ and FUS, help to resolve R-loops and maintain cellular homeostasis. Loss of paraspeckle components (for example, Che-1 and NEAT1) results in R-loop accumulation, leading to increased RNA–DNA hybrids and activation of cGAS–STING and RIG-1-mediated inflammatory signaling in multiple myeloma. **c** NF-κB-mediated R-loop accumulation in ATCL. Constitutive activation of NF-κB signaling promotes R-loop accumulation, which generates R-loop-derived inflammatory signals. This creates a positive feedback loop where R-loops further activate NF-κB signaling, leading to sustained inflammatory signaling and progression of ATCL.

R-loop-mediated inflammation in cancer is emerging as an important factor in disease progression and potential therapeutic targeting. Disruptions in R-loop homeostasis can trigger inflammatory responses across various types of blood cancer (Fig. 3). In hematological malignancies, such as acute myeloid leukemia (AML) and myelodysplastic syndrome, the loss or mutation of the DEAD-box helicase DDX41, an R-loop resolving helicase, leads to the accumulation of R-loops. While DDX41 was initially characterized as a DNA sensor that activates the STING pathway^90,91^, recent proximity proteomics studies have revealed its broader role in R-loop resolution and inflammatory response^92^ (Fig. 3a). RNA–DNA proximity proteomics, combined with quantitative mass spectrometry, has revealed important insights into proteins associated with R-loops in human cells. This study identified DDX41, a known tumor suppressor, as a critical regulator of R-loop dynamics, with particular importance in promoter regions of genes. DDX41 plays a vital role in maintaining genomic stability by preventing excessive R-loop formation. They have shown that DDX41 can directly unwind RNA–DNA hybrids in vitro, and it is particularly concentrated in promoter regions within living cells. When cells lose DDX41 function, R-loops begin to accumulate excessively, especially in promoter regions, cells undergo increased replication stress and there is a marked increase in double-strand DNA breaks. In addition, the loss of DDX41 leads to substantial changes in gene expression patterns, particularly affecting genes involved in inflammatory responses. These findings have implications for understanding certain types of blood cancers, specifically familial AML. People who inherit mutations that impair DDX41 function are more likely to develop AML in adulthood. The research suggests that this increased cancer risk may be due to the defective DDX41 leads to R-loop accumulation, which causes genomic instability, ultimately triggering inflammatory responses that may contribute to leukemia development.

Excessive R-loop accumulation triggers a complex inflammatory cascade that impacts cellular function and development^93^. Their study revealed that, when R-loops accumulate beyond normal levels, they initiate a sequence of cellular responses. Initially, this accumulation activates the DNA damage response, which leads to P53-dependent cell cycle arrest. This cellular stress response then triggers an inflammatory cascade that ultimately results in increased production of hematopoietic stem and progenitor cells. The inflammatory cascade involves the activation of innate immune pathways and the production of pro-inflammatory cytokines. These inflammatory signals have been shown to directly influence stem cell behavior and differentiation patterns. Specifically, the study demonstrated that R-loop-induced inflammation can alter the bone marrow microenvironment and affect hematopoietic development. The relationship between R-loop accumulation and increased hematopoietic stem and progenitor cell production highlights the important role of R-loops in regulating stem cell function and tissue homeostasis through inflammatory signaling pathways.

R-loop-mediated inflammation is also induced in multiple myeloma. AATF/Che-1 as a crucial regulator of R-loop accumulation and IFN responses in multiple myeloma^94^ (Fig. 3b). This study revealed that AATF/Che-1 specifically localizes to nuclear paraspeckles, where it plays a key role in preventing excessive R-loop formation. AATF/Che-1’s function in paraspeckles is notable as these nuclear bodies are known to be involved in various aspects of RNA metabolism. Within these structures, AATF/Che-1 acts to suppress R-loop accumulation, maintaining genomic stability. When AATF/Che-1 function is compromised, cells experience increased R-loop formation, which can lead to genomic instability. AATF/Che-1 not only results in R-loop accumulation but also triggers IFN activation, with higher levels of DNA–RNA hybrids in patients correlating with increased IFN pathway activation and poor prognosis.

Similar mechanisms of R-loop-mediated inflammation have been observed in adult T cell leukemia (ATL)^95^ (Fig. 3c). A study revealed that constitutive NF-κB activation, a hallmark of ATL, leads to R-loop accumulation and DNA damage. This creates a unique situation where NF-κB signaling, typically associated with inflammatory responses, promotes further inflammation through R-loop-mediated mechanisms. The accumulated R-loops cause genomic instability and DNA damage, which in turn selects for cells with deficiencies in nucleotide excision repair (NER). These NER-deficient cells show increased survival despite elevated R-loop levels, suggesting an adaptation mechanism that allows leukemia cells to thrive under conditions of chronic R-loop-induced stress. This demonstrates how cancer cells can evolve to exploit R-loop-mediated inflammation for their survival, creating a feed-forward loop between NF-κB activation, R-loop accumulation and inflammatory signaling in ATL progression.

## R-loop-mediated inflammation in breast and ovarian cancers

**Fig. 4:**
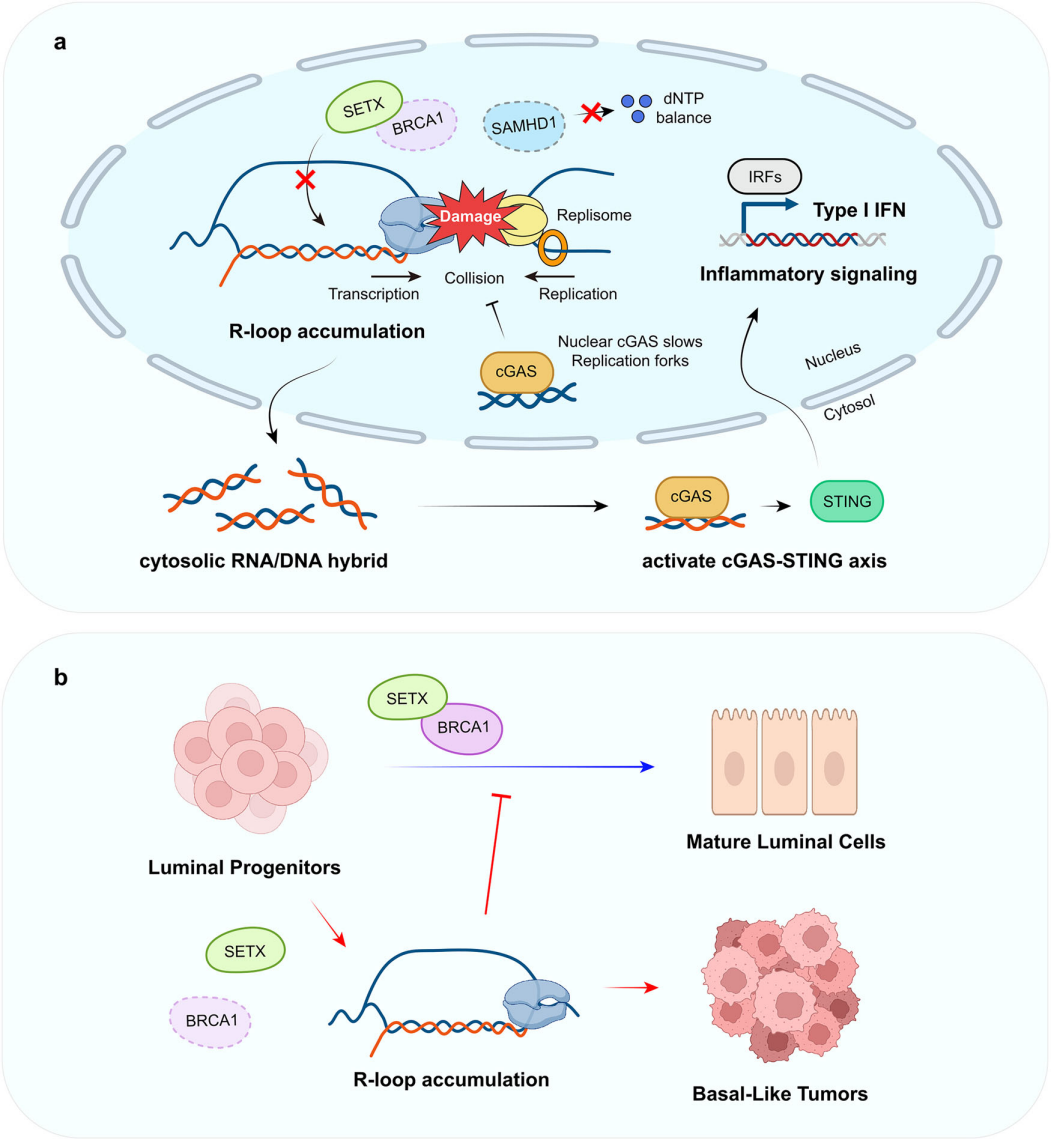
R-loop regulation and its role in inflammatory signaling in BRCA-deficient tumorigenesis. **a** Mechanisms of R-loop regulation and inflammatory signaling. SAMHD1 plays a critical role in maintaining genomic stability by regulating cellular dNTP pools and preventing R-loop formation. When SAMHD1 is deficient, the imbalance in dNTP levels impairs proper DNA replication and repair, leading to increased collisions between replication and transcription machinery, which promotes R-loop formation and accumulation. BRCA1 also functions as a key regulator of R-loop homeostasis by facilitating the recruitment of RNA processing factors and by interacting with SETX. In BRCA1-deficient cells, impaired R-loop resolution results in persistent R-loop structures and genome instability. These accumulated R-loops promote the release of cytosolic RNA–DNA hybrids, triggering cGAS–STING pathway activation, which induces type I IFN production and IRF-mediated inflammatory signaling. **b** R-loop accumulation in breast cancer development. In normal development, the BRCA1–SETX complex promotes the differentiation of luminal progenitors into mature luminal cells. Loss of BRCA1 function leads to R-loop accumulation in luminal progenitors, blocking normal differentiation and promoting the development of basal-like tumors.

Solid tumors also exhibit R-loop mediated inflammation. In breast and ovarian cancers associated with BRCA1/2 mutations, defects in R-loop resolution led to the accumulation of cytoplasmic R-loop byproducts^14^ (Fig. 4). BRCA1 facilitates the recruitment of SETX, an RNA–DNA helicase that specifically unwinds R-loops at transcription termination sites^32^. This BRCA1–SETX interaction is essential for preventing R-loop accumulation, particularly at regions of convergent transcription where R-loops are more likely to form. When these resolution pathways are compromised due to BRCA1 mutations, R-loops accumulate abnormally, leading to genomic instability, replication stress and DNA damage. The persistent R-loops in BRCA-deficient cells generate cytosolic nucleic acid species that activate PRRs such as cGAS and TLR3 (Fig. 4a). Recent findings by Chiang et al. show that the roles of R-loops in BRCA1-associated tumorigenesis^96^ (Fig. 4b). Using a transgenic mouse model where accumulated R-loops were removed in BRCA1-deficient mouse mammary epithelium through RNaseH1 overexpression, they demonstrated that R-loop removal did not influence the overall incidence of spontaneous BRCA1-associated mammary tumors. Rather, R-loop attenuation altered the cellular differentiation landscape, shifting the equilibrium between luminal progenitor and mature luminal cells. This resulted in a significant percentage of BRCA1-deficient tumors expressing ERα and progesterone receptor, unlike the typical triple-negative phenotype. These findings suggest that, instead of directly promoting tumorigenesis, R-loops primarily influence the breast cancer subtype by shaping the cell of origin for BRCA1-associated malignant transformation, affecting the tumor’s molecular profile and potentially its response to therapy. However, it remains unclear whether R-loop-mediated inflammation directly contributes to this phenotypic shift or if other mechanistic pathways are involved in shaping cell differentiation and subsequent tumor characteristics. Further studies are needed to elucidate the specific role of inflammatory signaling in determining breast cancer subtypes in the context of BRCA deficiency.

## R-loop-mediated inflammation in lung cancers

The loss of the tumor suppressor SAMHD1 in cancers similarly results in R-loop accumulation and IFN production^97,98^. Recent studies have highlighted the role of SAMHD1 in regulating R-loop accumulation^97^ and antitumor immune responses, particularly in lung adenocarcinoma^98^. Research has shown that silencing SAMHD1 leads to increased DNA damage, which cooperates with radiotherapy to enhance antitumor immunity. When SAMHD1 is suppressed, the accumulated R-loops may trigger the IFI16–STING pathway, a key mediator of innate immune responses. The activation of this pathway is important in the context of radiotherapy, where DNA damage and R-loop formation are already enhanced. The mechanistic pathway begins with SAMHD1 silencing, which leads to R-loop accumulation, while radiotherapy further increases DNA damage and R-loop formation. These accumulated nucleic acid structures are recognized by IFI16, which then activates the STING pathway, ultimately leading to type I IFN production and enhanced antitumor immune responses. This cooperative effect between SAMHD1 silencing and radiotherapy represents a potential therapeutic strategy for improving cancer treatment outcomes. The enhanced activation of the IFI16–STING pathway through R-loop accumulation suggests that targeting SAMHD1 could be particularly effective in combination with conventional radiotherapy, leading to stronger antitumor immune responses in patients with lung adenocarcinoma.

## R-loop-mediated inflammation in liver cancers

The study by Waqar Arif et al. demonstrates a critical link between splicing dysregulation and inflammation in the context of liver disease that may have important implications for cancer pathogenesis^99^. SRSF1 deficiency in hepatocytes was shown to promote R-loop formation, which triggered DNA damage and subsequent global inhibition of mRNA transcription and protein synthesis. The researchers observed that SRSF1-knockout mice developed severe hepatic inflammation, fibrosis and cell death characteristic of nonalcoholic steatohepatitis (NASH). Importantly, the study established that R-loop accumulation was the primary driver of this inflammatory phenotype. This work provides compelling evidence that disruption of RNA splicing factors can promote genomic instability via R-loop formation, subsequently activating inflammatory pathways that may contribute to an inflammatory microenvironment conducive to cancer development and progression.

## R-loop-mediated inflammation in SWI–SNF complex mutated cancer

**Fig. 5:**
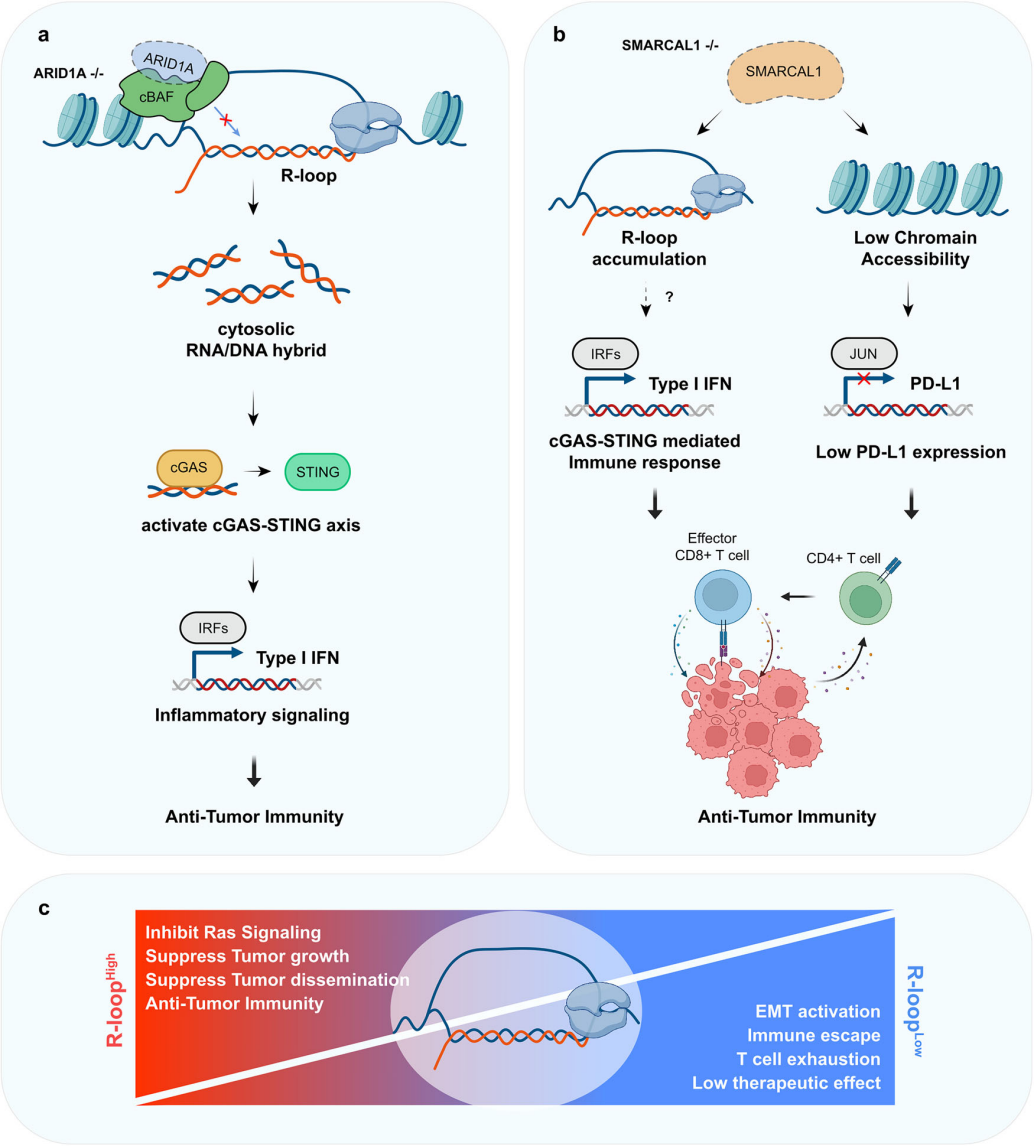
R-loop regulation in antitumor immunity. **a** ARID1A-deficient cancer cells show enhanced R-loop formation. Loss of ARID1A, a component of the cBAF complex, leads to increased R-loop accumulation. These R-loops generate cytosolic RNA–DNA hybrids that activate the cGAS–STING pathway, triggering type I IFN production through IRF activation, ultimately promoting antitumor immunity. **b** SMARCAL1 deficiency affects both R-loop accumulation and immune checkpoint expression. Loss of SMARCAL1 leads to two parallel effects: R-loop accumulation that activates cGAS–STING-mediated immune responses and reduced chromatin accessibility that decreases PD-L1 expression through impaired JUN-mediated transcription. These combined effects enhance antitumor immunity. **c** The R-loop balance model in cancer progression. High R-loop levels (red) can inhibit Ras signaling, suppress tumor growth and dissemination, and promote antitumor immunity. Conversely, low R-loop levels (blue) are associated with EMT activation, immune escape, T cell exhaustion and reduced therapeutic efficacy.

The SWItch/Sucrose Non-Fermentable (SWI–SNF) complex is a crucial chromatin remodeling complex that plays a vital role in gene regulation, DNA repair and cell differentiation^100^. Mutations in the components of this complex have been increasingly recognized as important drivers in various types of cancer. Mutations in SWI–SNF subunits are found in approximately 20% of all human cancers^101,102^. Among SWI–SNF complexes, the canonical BAF (cBAF) complex is the most altered variant in cancer^103^. This complex is characterized by the inclusion of specific subunits: ARID1A, ARID1B and DPF2^104^. Within the cBAF complex, *ARID1A* is the subunit gene that undergoes mutations most frequently^105^. It is also one of the most often mutated tumor suppressor genes in human cancers. The mutations in *ARID1A* are typically inactivating, leading to a loss of protein expression. These mutations are found in 8–60% of various cancers, including endometrial, ovarian clear cell, colon, gastric, liver and pancreatic cancers^106,107^. Maxwell et al. provide mechanistic insights into how ARID1A deficiency promotes antitumor immunity and enhances immune checkpoint blockade responses through R-loop-mediated activation of the STING-type I IFN pathway^71^ (Fig. 5a). The authors demonstrate that ARID1A loss leads to the accumulation of R-loops, which give rise to cytosolic DNA species. These cytosolic DNA fragments activate STING-dependent type I IFN signaling, inducing an *ARID1A-IFN* gene expression signature that promotes antitumor immunity. This mechanism was shown to be sufficient to induce antitumor immunity and enhance immune checkpoint blockade responses in murine tumor models. Importantly, the study reveals that ARID1A-deficient tumors exhibit increased infiltration of CD8^+^ T cells with enhanced functionality. Single-cell RNA sequencing analysis of tumor-infiltrating CD8^+^ T cells showed a shift from naive-like states toward more effector-like and exhausted states, which are associated with key antitumor functions such as self-renewal and cytotoxicity. The ARID1A-IFN signature was also found to be upregulated in these T cells, potentially contributing to their enhanced antitumor activity. The researchers observed increased expression of chemokines and antigen presentation-related genes in ARID1A-deficient tumors, facilitating immune cell recruitment and T cell activation. This alteration in the tumor microenvironment probably contributes to the enhanced antitumor immune response observed in ARID1A-deficient cancers. Intriguingly, the authors found that pharmacological inhibition of the SWI–SNF complex could phenocopy *ARID1A* mutation with respect to induction of the ARID1A-IFN signature. This observation suggests a potential therapeutic strategy to convert immunologically ‘cold’ tumors into more immune-infiltrated ‘hot’ tumors by inducing R-loop-driven ARID1A-IFN induction through SWI–SNF inhibition.

## Therapeutic implication of R-loop mediated inflammation in tumor microenvironment

### SMARCAL1 as a dual regulator in cancer immunity

SWI/SNF-related matrix-associated actin-dependent regulator of chromatin subfamily A-like protein 1 (SMARCAL1) has been found to play a pivotal role in regulating R-loop homeostasis. SMARCAL1 is an SNF2-family DNA translocase whose activity is essential for two cellular functions: replication fork reversal and R-loop unwinding^108,109^. Leuzzi et al. demonstrated that SMARCAL1 concurrently regulates both innate immune signaling and immune checkpoint expression in cancer cells^110^ (Fig. 5b). The first mechanism involves SMARCAL1’s role in DNA damage control. SMARCAL1 functions to limit endogenous DNA damage in cancer cells, thereby suppressing the activation of cGAS–STING-dependent signaling during cancer cell growth. This study did not show the origin of endogenous DNA damage, but the accumulation of R-loops might be the source of endogenous DNA damage and cytoplasmic DNA in SMARCAL1-depleted cells. The cGAS–STING pathway is a crucial innate immune sensor that typically recognizes cytoplasmic DNA as a danger signal and triggers antitumor immune responses. By limiting DNA damage, SMARCAL1 helps cancer cells to avoid detection by this immune surveillance mechanism. The second mechanism reveals SMARCAL1’s direct involvement in immune checkpoint regulation. SMARCAL1 collaborates with JUN, a member of the AP-1 transcription factor family, to maintain chromatin accessibility at specific regulatory elements that control PD-L1 transcription. Through this mechanism, SMARCAL1 actively promotes PD-L1 expression in cancer cells. PD-L1, as a critical immune checkpoint molecule, plays a crucial role in cancer cells’ ability to evade immune surveillance by suppressing T cell responses. When SMARCAL1 function is lost, cancer cells experience changes in their immune interactions. The loss of SMARCAL1 impairs the cells’ ability to effectively induce PD-L1 in response to genomic instability. This impairment leads to enhanced antitumor immune responses. Furthermore, tumors lacking SMARCAL1 show increased sensitivity to immune checkpoint blockade therapy, as demonstrated in a mouse melanoma model. These findings have implications for cancer immunotherapy. By identifying SMARCAL1 as a dual regulator that simultaneously suppresses innate immune signaling and promotes immune checkpoint expression, this research suggests that targeting SMARCAL1 could provide a multifaceted approach to cancer treatment.

## R-loops in MYCN-amplified neuroblastoma and therapeutic implications

Further insights into the relationship between R-loops and cancer pathogenesis come from studies on MYCN-amplified neuroblastoma^111^. Research has demonstrated that MYCN amplification, a genetic alteration associated with aggressive neuroblastoma, leads to increased transcriptional stress and R-loop formation. The accumulation of R-loops in these cells contributes to DNA damage and activation of inflammatory pathways, creating a pro-tumorigenic microenvironment.

Interestingly, the therapeutic strategy involving combined inhibition of Aurora-A and ATR kinases has shown promise in treating MYCN-amplified neuroblastoma. This approach not only destabilizes the MYCN protein but also compromises the DNA damage response mechanisms that neuroblastoma cells rely on to survive R-loop-induced genomic stress. The dual targeting creates a synthetic lethal interaction that leads to tumor regression in preclinical models. This therapeutic strategy indirectly addresses the R-loop-mediated inflammatory processes by targeting the upstream drivers and downstream consequences of R-loop formation.

## R-loop mediated immune evasion and cell comminutions

R-loops exert profound effects on immune evasion and intercellular signaling networks within the tumor microenvironment (Fig. 5c). Zhang et al. demonstrated through single-cell RNA sequencing of lung adenocarcinoma that cancer cells with low R-loop scores exhibit reduced expression of tumor-associated antigens and MHC molecules, coupled with upregulation of immunosuppressive factors^112^. This transcriptional reprogramming directly contributes to immune evasion, as indicated by increased CD4^+^ and CD8^+^ T cell dysfunction and elevated NK cell exhaustion scores in tumors with low R-loop burden. In the context of cellular communication, high R-loop scores positively correlate with enhanced expression of chemokines (CXCL16 and CXCL2) and costimulatory molecules (TNFSF12, ICAM1 and TNFRSF14) in malignant cells. Conversely, low R-loop scores promote communication between tumor cells and T cells through co-inhibitory pathways, contributing to T cell exhaustion. The mechanistic link between R-loops and intercellular communication was further confirmed by FANCI-knockdown experiments, which altered the R-loop landscape and impacted genes involved in both Ras and MAPK signaling pathways. These findings highlight the multifaceted role of R-loops in orchestrating both immune evasion mechanisms and intercellular communication networks that collectively shape tumor progression.

## Concluding remarks and future perspectives

The study of R-loop-mediated inflammation in cancer opens numerous exciting opportunities while presenting challenges for future research. As our understanding of these complex nucleic acid structures continues to deepen, several critical questions remain unanswered in the field. We still need to unravel how cells maintain the delicate balance between beneficial and harmful R-loops during tumor development. The specific mechanisms through which R-loops trigger immune responses, and how cancer cells manipulate these responses, require further investigation. Moreover, understanding the diverse roles of R-loops across different cancer types and their contribution to tumor heterogeneity remains a crucial area for exploration.

Technological advancements are providing new opportunities to study R-loops with unprecedented detail. The emergence of single-cell sequencing technologies allows researchers to examine the heterogeneity of R-loop formation and its effects on cellular responses at an individual cell level. There is also a pressing need for advanced imaging techniques that can track R-loop dynamics in real time within living cells. Developing more specific tools for manipulating R-loop levels in vivo will be essential for understanding their biological roles and therapeutic potential. The translation of R-loop research from laboratory findings to clinical applications faces several challenges. Developing specific therapeutic agents that can effectively target R-loop formation or processing while minimizing off-target effects remains a major hurdle. Identifying reliable biomarkers for R-loop-associated inflammation in cancer will be crucial for patient stratification and treatment monitoring. Understanding how potential R-loop-targeting therapies might interact with existing cancer treatments is also essential for successful clinical implementation. Looking ahead, several promising research directions emerge in the field. Investigating cell-type-specific responses to R-loop formation could reveal new therapeutic opportunities. Understanding the complex relationship between R-loops and tumor immunology may lead to novel immunotherapy approaches. The development of R-loop-targeted therapeutic strategies, along with exploring their potential as prognostic or diagnostic markers, represents an exciting frontier in cancer research.

Furthermore, studying the role of R-loops in treatment resistance could provide insights into improving cancer therapy outcomes. Success in these areas will require continued technological innovation and interdisciplinary collaboration. As we advance our understanding of R-loop biology in cancer, these insights will ultimately contribute to more effective therapeutic strategies for cancer treatment.
